# Genes Involved in Type 1 Diabetes: An Update

**DOI:** 10.3390/genes4030499

**Published:** 2013-09-16

**Authors:** Marina Bakay, Rahul Pandey, Hakon Hakonarson

**Affiliations:** 1 Center for Applied Genomics, Children’s Hospital of Philadelphia, Philadelphia, PA 19104, USA; E-Mails: bakay@email.chop.edu (M.B.); pandeyr@email.chop.edu (R.P.); 2 Department of Pediatrics, The Perelman School of Medicine at the University of Pennsylvania, Philadelphia, PA 19104, USA

**Keywords:** Type 1 Diabetes (T1D), genome-wide association studies (GWAS), immune system, susceptibility loci, natural killer (NK) cells, pancreatic β-cells

## Abstract

Type 1 Diabetes (T1D) is a chronic multifactorial disease with a strong genetic component, which, through interactions with specific environmental factors, triggers disease onset. T1D typically manifests in early to mid childhood through the autoimmune destruction of pancreatic β cells resulting in a lack of insulin production. Historically, prior to genome-wide association studies (GWAS), six loci in the genome were fully established to be associated with T1D. With the advent of high-throughput single nucleotide polymorphism (SNP) genotyping array technologies, enabling investigators to perform high-density GWAS, many additional T1D susceptibility genes have been discovered. Indeed, recent meta-analyses of multiple datasets from independent investigators have brought the tally of well-validated T1D disease genes to almost 60. In this mini-review, we address recent advances in the genetics of T1D and provide an update on the latest susceptibility loci added to the list of genes involved in the pathogenesis of T1D.

## 1. Introduction

Type 1 Diabetes (T1D) is a chronic multifactorial disease with a strong genetic component. It arises as a consequence of autoimmune destruction of pancreatic β-cells, resulting in insufficient insulin production. The prevalence of diabetes is increasing worldwide [1]. According to the International Diabetes Federation (IDF), the worldwide prevalence of diabetes mellitus in 2011 was 366 million, and is predicted to reach 552 million by 2030 [2]. T1D represents approximately 10% of these patients and is most prevalent in populations of European ancestry [3,4]. There is about 3% increase in the incidence of T1D annually, lending further support to complex gene environment interactions in the pathogenesis of T1D [3]. While cumulative evidence supports a strong genetic component associated with T1D, epidemiological data show wide differences in geographic prevalence with populations of European ancestry having the highest presentation rate. T1D also has high concordance among monozygotic twins (33% to 42%) [5], and the disease runs strongly in families with siblings risk being approximately 10 times greater than in the general population [6]; this is in clear contrast to the “less genetic” type 2 diabetes, where the sibling risk ratio is relatively modest at 3.5 [7].

T1D develops at all ages and occurs through the autoimmune destruction of pancreatic β-cells with resulting lack of insulin production. The immune system participates in β-cell destruction through several of its components including CD4^+^ and CD8^+^ T cells, natural killer (NK) cells, B lymphocytes, macrophages, dendritic cells (DC), and antigen-presenting cells (APCs). Studies in human and animal models have shown that both innate and adaptive immune responses participate in disease pathogenesis, possibly reflecting the multifactorial nature of this autoimmune disorder.

In this review, we will provide an updated summary of genome-wide association studies (GWAS) including recent meta-analyses and discuss the latest associated regions added to the growing repertoire of gene networks predisposing to T1D.

## 2. Genetic Component in Type 1 Diabetes

**Figure 1:**
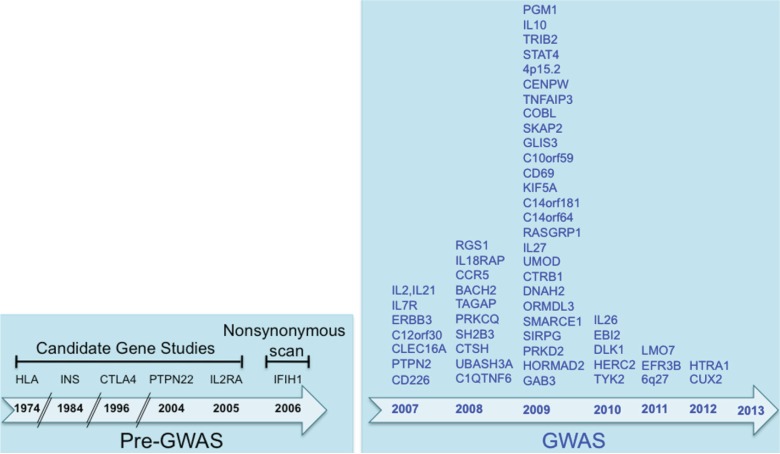
The Type 1 Diabetes loci described to date: a timeline. The susceptibility loci are presented by the year they were first implicated in T1D.

The risk of developing T1D is determined by a complex interaction between multiple genes and environmental factors. The discovery of T1D susceptibility genes started as early as 1974 with five genes discovered, using family and candidate gene approaches. The advent of GWAS led to flurry of novel genes associated with T1D reaching the excess of 40 by 2006, and 60 by 2012 as depicted in Figure 1. It is clear now that many of these genes are novel and were not on any investigators’ radars when they were designing candidate gene studies in the past.

### 2.1. Before Genome-Wide Association Studies

Historically, prior to GWAS, only six loci were fully established to be associated with T1D. The human leukocyte antigen (HLA) region on chromosome 6p21 was the first known candidate to be strongly associated with T1D in the 1970s [8,9,10]. This cluster of homologous cell-surface proteins is divided into class I (A, B, C) and class II (DP, DQ, RD). The HLA genes encode highly polymorphic proteins, which are essential in self- *versus* non self-immune recognition. The class I molecules are ubiquitously expressed and present intracellular antigen to CD8^+^ T cells. Class II molecules are expressed mainly on professional APCs: DCs, macrophages, B lymphocytes and thymus epithelium. Class II molecules are composed of A and B chains, and present antigens to CD4^+^ T cells, which promote inflammation by secreting cytokines upon recognition of their specific targets. Approximately half of the genetic risk for T1D is conferred by the genomic region harboring the HLA class II genes (primarily HLA-DRB1, -DQA1 and -DQB1 genes). In 1984, insulin (INS) gene encoded on chromosome 11p15 was identified as the second loci linked with T1D [11]. In 1996, the cytotoxic T-lymphocyte-associated protein 4 (CTLA4) gene encoded on chromosome 2q33 was recognized as the third loci [12]. Another case-control study in 2004 reported a protein tyrosine phosphatase, non-receptor type 22 (PTPN22), gene encoded on chromosome 1p13 to be associated with susceptibility to T1D [13]. Vella *et al.*, 2005 reported interleukin 2 receptor alpha (IL2RA) gene as the fifth T1D loci on chromosome 10p15 [14]. In 2006, Smyth *et al.* identified the interferon-induced with helicase C domain 1 (IFIH1) gene on chromosome 2q24.3 as the sixth candidate to be strongly associated with T1D through genotyping of only 6,500 non-synonymous SNPs genome wide [15]. This study was a precursor to the first GWAS approach.

### 2.2. GWAS of T1D

The advent of GWAS in the mid-2000s has changed the situation dramatically, increasing the pace and efficiency of discovery for the T1D associated loci by a factor of ten. The critical platform for this work was laid by the HapMap project [16,17]. The GWAS approach was made possible by the development of high-density genotyping arrays. It has been shown that the genome is laid out in discrete linkage disequilibrium (LD) blocks, with limited haplotype diversity within each of these blocks. Therefore, a minimal set of single nucleotide polymorphisms (SNPs) can detect almost all common haplotypes present, thus improving genotyping accuracy and reducing the cost. As a result, these technologies enable us and others to perform GWA studies in search of the remaining T1D loci, the outcomes of which are outlined below.

The first full-scale GWAS for T1D were published simultaneously by our group [18] and by the Wellcome Trust Case-Control Consortium (WTCCC) [19]. We examined a large pediatric cohort of European descent using the Illumina HumanHap 550 BeadChip platform. The design involved 561 cases, 1,143 controls, and 467 triads in the discovery stage, followed by a replication effort in 939 nuclear families. In addition to finding the “usual” suspects, including an impressive 392 SNPs capturing the very strong association across the major histocompatibility complex (MHC), we identified significant association with variation at the KIAA0350 gene, which we replicated in an additional cohort. The WTCCC study investigated seven common complex diseases including T1D by genotyping 2,000 cases and 3,000 controls with ~500,000 SNPs using the Affymetrix GeneChip, and reported a number of novel T1D loci, including the KIAA0350 genomic region [19]. Todd *et al.*, 2007 replication study confirmed these findings in 4,000 cases, 5,000 controls, and 3,000 T1D families [20]. In a separate replication effort we elected to fast-track 24 SNPs at 23 distinct loci and established association to the 12q13 region with a combined *p*-value of 9.13 × 10^−10^ [21], previously reported by the WTCCC [19] and Todd *et al.* [20]. The 250-kb LD block on 12q13 region harbors several genes, including ERBB3, RAB5B, SUOX, RPS26, and CDK2. Additional laboratory studies are needed to identify both the corresponding genes and the causative variants for this locus. Later the same year, Concannon *et al.* reported an association between SNP at the UBASH3A locus on 21q22.3 and T1D by using SNP genotyping data from a linkage study of affected sib pairs in nearly 2,500 multiplex families [22]. UBASH3A (previously known as T-cell ubiquitin ligand [TULA] and suppressor of T-cell signaling 2 [Sts-2]) is expressed predominantly in T cells. It interacts with c-CBL through its SH3 domain and binds to ubiquitin and ubiquitylated proteins via its UBA domain [23]. UBASH3A protein product similar to PTPN22 interacts with c-CBL, but UBASH3A directly downregulates some of the same protein tyrosine kinases by dephosphorylation [24]. Follow-up of 1715 SNPs from the WTCCC genome-wide association study in T1D families confirmed UBASH3A as a susceptibility gene [25]. A recent study reported UBASH3A to be an independent predictor of persistent islet autoimmunity and T1D in children, including those free of family history of T1D but carrying the HLA-DR3/4, DQB1*0302 genotype. UBASH3A may prove useful in T1D risk prediction and pre-screening of the general population children for clinical trials [26].

### 2.3. Meta-Analyses of T1D GWAS Datasets

In order to get the most from GWAS and to increase the statistical power researchers carried out meta-analyses using datasets from different investigative groups. First meta-analysis was performed by combining the T1D datasets from the Wellcome Trust Case Control Consortium [19] and the Genetics of Kidneys in Diabetes (GoKind) study [27,28], plus control data derived from the National Institute of Mental Health. This study confirmed associations for PTPN22, CTLA4, MHC, IL2RA, 12q13, 12q24, CLEC16A, and PTPN2 [29]. The SNPs with lowest nominal *p*-values were taken forward for further genotyping in an additional British cohort of approximately 6,000 cases, 7,000 controls, and 2,800 families. As a result, the IL2-IL21 association strengthened further and they found strong evidence for four additional loci: a 6q15 region harboring BACH2; a 10p15 region harboring the protein kinase C, theta gene (PRKCQ); a 15q24 region harboring nine genes including cathepsin H (CTSH) and 22q13 harboring the C1q and tumor necrosis factor-related protein 6 (C1QTNF6) and somatostatin receptor 3 (SSTR3) genes [29]. Study of polymorphisms in 4q27, 10p15, and 22q13 regions in autoantibodies stratified type 1 diabetes patients further confirmed IL2 association in pediatric patients and individuals with late onset of T1D [30]. Additional studies are required to elucidate the culprit genes and their mechanism at the 15q24 and 22q13 loci.

Meta-analysis by T1DGC [31] provided evidence of T1D association for 41 distinct genomic locations (*p* < 10^−6^) by using datasets from WTCCC [19], the GoKind study [28], and controls and family sets from Type 1 Diabetes Genetics Consortium (T1DGC). The study confirmed a number of previously reported associations [32,33,34] and discovered 22 novel, of which 18 regions were replicated (*p* < 5 × 10^−8^) and four additional regions provided nominal evidence of replication (*p* < 0.05). The meta-analysis observed association to 1q32.1 (which harbors the immunoregulatory interleukin genes IL10, IL19 and IL20), 9p24.2 contains only Glis family zinc finger protein 3 (GLIS3), which was first suggested by us in [35], 12p13.31 which harbors a number of immunoregulatory genes including CD69 and 16p11.2 harboring IL27. Our *in silico* replication efforts [36] further confirmed the associations previously reported by the T1DGC [31]. The entire Barrett *et al.* study was later replicated in 2012 by T1DGC to exclude the possibility that any of the 18 loci were false-positives due to population stratification. Seventeen of the 18 susceptibility loci reached nominal levels of significance (*p* < 0.05) in the expanded family collection, with 14q24.1 just falling short (*p* = 0.055) [37]. All susceptibility loci had consistent direction of effects with the original study.

To identify additional genetic loci for T1D susceptibility, we examined associations in the largest meta-analysis to date between the disease and ~2.54 million SNPs in a combined cohort of 9,934 T1D cases and 16,956 controls [38]. Targeted follow-up of 53 SNPs in 1,120 affected trios uncovered three new loci associated with T1D that reached genome wide significance. The most significantly associated SNP (rs539514, *p* = 5.66 × 10^−11^) resided in an intronic region of the LMO7 (LIM domain only 7) gene on 13q22. The second most significantly associated SNP (rs478222, *p* = 3.50 × 10^−9^) resided in an intronic region of the EFR3B (protein EFR3 homolog B) gene on 2p23; however the region of linkage disequilibrium is approximately 800 kb and harbors additional multiple genes, including NCOA1, C2orf79, CENPO, ADCY3, DNAJC27, POMC, and DNMT3A. The third most significantly associated SNP (rs924043, *p* = 8.06 × 10^−9^) was in an intergenic region on 6q27, where the region of association is approximately 900 kb and harbors additional genes including WDR27, C6orf120, PHF10, TCTE3, C6orf208, LOC154449, DLL1, FAM120B, PSMB1, TBP, and PCD2. These latest associations add to the growing repertoire of gene networks predisposing to T1D. Table 1 summarizes all T1D associated loci reported to date.

**Table 1:** T1D susceptibility loci identified to date.

Reference	Study Type		Main Findings	Sample Size	Replication Sample Size	Ethnic Group
Hakonarson *et al.*, 2007 [18]	GWAS		HLA-DRB1, HLA-DQA2, CLEC16A, INS, PTPN22	467 trios, 561 cases, 1,143 controls	2,350 individuals in 549 families; 390 trios	European ancestry
WTCCC 2007 [19]	GWAS		HLA-DRB1, INS, CTLA4, PTPN22, IL2RA, IFIH1, PPARG, KCNJ11, TCF7L2	1,963 cases, 2,938 controls	see Todd *et al.*, 2007	European, British
Todd *et al.*, 2007 [20]	GWAS		PHTF1-PTPN22, ERBB3, CLEC16A, C12orf30	see WTCCC 2007	2,997 trios, 4,000 cases, 5,000 controls	European, British
Hakonarson *et al.*, 2008 [21]	GWAS		SUOX-IKZF4	467 trios, 561 cases, 1,143 controls	549 families, 364 trios	European ancestry
Concannon *et al.*, 2008 [22]	GWAS		INS, IFIH1, CLEC16A, UBASH3A	2,496 families	2,214 trios, 7,721 cases, 9,679 controls	European ancestry
Cooper *et al.*, 2008 [29]	GWAS meta-analysis		PTPN22, CTLA4, HLA, IL2RA, ERRB3, C12orf30, CLEC16A, PTPN2	3,561 cases, 4,646 controls	6,225 cases, 6,946 controls, 3,064 trios	European ancestry
Grant *et al.*, 2009 [35]	GWAS		EDG7, BACH2, GLIS3, UBASH3A, RASGRP1	563 cases, 1,146 controls, 483 case-parents trios	636 families, 3,303 cases, 4,673 controls	European ancestry
Awata *et al.*, 2009 [39]	TaqMan genotyping		ERBB3, CLEC16A	735 cases, 621 controls	−	Japanese
Zoledziewska *et al.*, 2009 [4^40^]	TaqMan genotyping		CLEC16A	1037 cases, 1706 controls	−	European, Sardinian
Fung *et al.*, 2009 [33]	TaqMan genotyping		STAT4, STAT3, ERAP1, TNFAIP3, KIF5A/PIP4K2C	8010 cases, 9733 controls	−	European, British
Wu *et al.*, 2009 [41]	TaqMan genotyping		CLEC16A	205 cases, 422 controls	−	Han Chinese
Barrett *et al.*, 2009 [31]	GWAS meta-analysis		MHC, PTPN22, INS, C10orf59, SH2B3, ERBB3, CLEC16A, CTLA4, PTPN2, IL2RA, IL27, C6orf173, IL2, ORMDL3, GLIS3, CD69, IL10, IFIH1, UBASH3A, COBL, BACH2, CTSH, PRKCQ, C1QTNF6, PGM1	7,514 cases, 9,045 controls	4,267 cases, 4,670 controls, 4,342 trios	European
Wallace *et al.*, 2010 [42]	GWAS meta-analysis		DLK1, TYK2	7,514 cases, 9,045 controls	4,840 cases, 2,670 controls, 4,152 trios	European ancestry
Wang *et al.*, 2010 [43]	GWAS		PTPN22, IL10, IFIH1, KIAA0746, BACH2, C6orf173, TAGAP, GLIS3, L2R, INS, ERBB3, C14orf181, IL27, PRKD2, HERC2, CLEC16A, IFNG, IL26	989 cases, 6,197 controls	−	European ancestry
Reddy *et al.*, 2011 [44]		TaqMan genotyping	PTPN22, INS, IFIH1, SH2B3, ERBB3, CTLA4, C14orf181, CTSH, CLEC16A, CD69, ITPR3, CENPW, SKAP2, PRKCQ, RNLS, IL27, SIRPG, CTRB2	1,434 cases, 1,864 controls	−	European ancestry, southeast USA
Bradfield *et al.*, 2011 [38]		GWAS meta-analysis	LMO7, EFR3B, 6q27, TNFRSF11B, LOC100128081, FOSL2	9,934 cases, 16,956 controls	1,120 trios	European ancestry
Asad *et al.*, 2012 [45]		Genotyping andsequencing	HTR1A, RFN180	424 families, 3,078 cases, 1,363 controls	−	European, Scandinavians
Huang *et al.*, 2012 [46]		Genomes-based imputation	CUX2, IL2RA	16,179 individuals	−	European ancestry

### 2.4. Immune Components in T1D

The immune system is well organized and well regulated with a basic function of protecting the host against pathogens. This places the immune system in a vital position between healthy and diseased states of the host. Its protective task is regulated by a complex regulatory mechanism involving a diverse army of cells and molecules of humoral and cellular factors working in concert to protect the body against invaders. Our immune system has two components: innate and adaptive. Innate immunity is comprised of physical, chemical, and microbiological barriers to the entry of antigen, and the elements of immune system (DC, macrophages, mast cells, NK cells, neutrophils, monocytes, complements, cytokines, and acute phase proteins), which provide immediate host defense. Adaptive immunity is the hallmark of the immune system of higher animals with T and B cells as the key cellular players that provide more specific life-long immunity [47].

**Figure 2:**
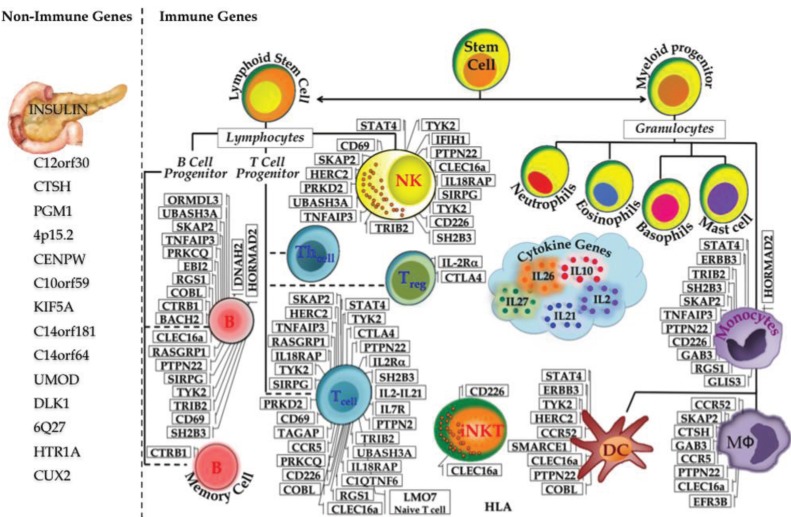
Immune and Non-immune T1D genes. The discovery of T1D susceptibility genes started as early as 1974, with six T1D genes identified by 2006. The advent of GWAS led to flurry of novel genes associated with T1D reaching the excess of 40 by 2009 and almost 60 by 2012.

In T1D this system breaks down: insulin-producing β-cells are subjected to specific attack by the host immune system. To better understand the etiology of T1D for prevention and cure, a plethora of research has been done to link the systematic destruction of β-cells and the role of the immune system, however the exact mechanism of T1D pathogenesis is not completely elucidated. Linkage studies in the 1970s revealed MHC as the first key contributor to T1D susceptibility [8,9,10]. Further linkage analysis and candidate gene association studies uncovered additional T1D loci. Starting in 2007, GWAS has increased the number of loci associated with T1D to almost 60 [38]. As T1D is an immune-mediated disorder the majority of candidate genes exert their functions in immune cells. In Figure 2, we have made an attempt to classify all 59 T1D susceptibility loci/genes in keeping with their predominant function of either non-immune (14) *vs.* immune (45). However, recent studies indicate that many T1D candidate genes are also expressed in human islets suggesting that functions are not restricted to immune cells, but also play roles in the islets and β cells [48]. The functional aspects of some of the most interesting genes or biological pathways are discussed below.

**Figure 3:**
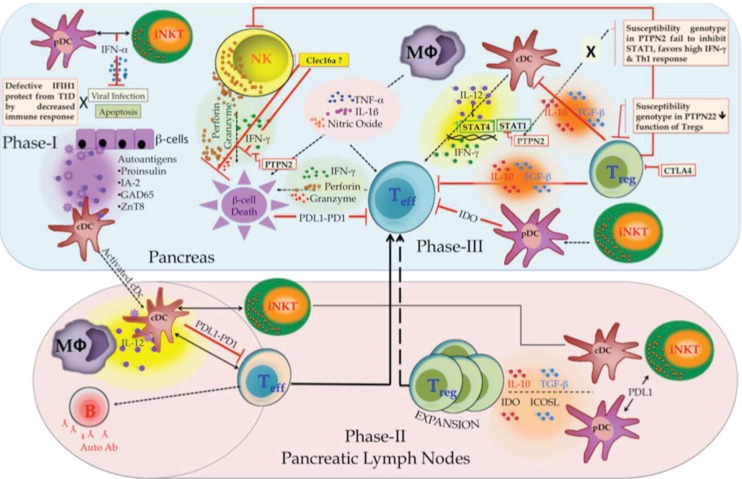
Pathogenesis model of T1D involves complex interactions between innate and adaptive immune cell types.

The complex crosstalk between innate and adaptive immune cells is broadly categorized in three phases, which results in the development or the prevention of T1D and is illustrated in Figure 3 as a hypothetical model.

Phase I (the initiation phase of T1D) involves β-cell death and APC activation. It takes place in the pancreas where conventional dendritic cells (cDCs) capture and process β-cell antigens. Natural cell death (apoptosis) or viral infection can lead to β-cell death. Antiviral responses are mediated by invariant natural killer T (iNKT) cells; crossplay between iNKT, and plasmacytoid DCs (pDCs) controls viral replication thus prevents subsequent inflammation, tissue damage, and downregulation of T1D pathogenesis [49].

Phase II (the expansion phase) involves expansion of self-antigens and specific T cells. Migration of activated cDCs to the draining lymph node primes pathogenic islet antigen-specific T cells. This activation is promoted by macrophages through IL12 secretion. B cells present β-cell antigen to diabetogenic T cells and secrete autoantibodies in response. The activation of islet antigen-specific T cells can be inhibited by cDCs through engagement of programmed cell death ligand 1 (PDL1). iNKT cells can further promote the recruitment of tolerogenic cDCs and pDCs. These DCs promote expansion of regulatory T (TReg) cells through the production of indoleamine 2,3-dioxygenase (IDO), IL10, transforming growth factor-β (TGFβ) and inducible T cell co-stimulator ligand (ICOSL) [50].

Phase III (immune cell crosstalk) occurs in the pancreas where β-cell can be killed by diabetogenic T cells (adaptive component) and NK cells (innate component) through the release of interferon-γ (IFNγ), granzymes, and perforin, as well as by macrophages through the production of tumor necrosis factor (TNF), IL-1β and nitric oxide (NO). IL12 produced by cDCs sustains the effector functions of activated diabetogenic T cells and NK cells. TReg cells that inhibit diabetogenic T cells and innate immune cells through IL10 and TGFβ can prevent β-cell damage. Tolerogenic pDCs stimulated by iNKT cells could also control diabetogenic T cells through IDO production. Lastly, β-cells can inhibit diabetogenic T cells by expressing PDL1 and escape the cell death [50,51].

There is increasing evidence that innate cells play critical roles in T1D onset. In our 2007 GWAS we identified CLEC16A as a novel T1D susceptibility gene [18]. CLEC16A is almost exclusively expressed in immune cells. As CLEC16A SNPs were associated with T1D protection and some of the highest expression of CLEC16A was identified in NK cells, we hypothesize that CLEC16A may function in NK cells to restrain secretory functions including cytokine release and cytotoxicity after activation (Figure 3).

### 2.5. Insights from T1D Specific Loci

Four decades of intensive studies have discovered nearly 60 T1D susceptibility loci; however the exact mechanisms by which associated loci confer T1D susceptibility remain elusive and require in depth characterization. Several novel T1D susceptibility genes are discussed below.

#### 2.5.1. CLEC16A (16p13)

Our 2007 GWAS in a large pediatric cohort of European descent identified CLEC16A as a novel T1D susceptibility gene within a 233-kb linkage disequilibrium block on chromosome 16p13. Three common non-coding variants of the CLEC16A gene (rs2903692, rs725613, and rs17673553) reached genome-wide significance for association with T1D [18]. Importantly, the allele of CLEC16A linked to protection from T1D was also associated with higher levels of CLEC16A expression in NK cells [18]. The C-type lectin domain family 16, member A (CLEC16A) gene encodes protein with C-type lectin domain structure, which makes it potentially related to the immune response [52]. It is established that C-type lectins function both as adhesion and pathogen recognition receptors (PPRs) [53]. In addition, CLEC16A is almost exclusively expressed in immune cells including DCs, B lymphocytes, and NK cells.

The 2007 WTCCC study independently discovered CLEC16A (formally known as KIAA0350) as a T1D susceptibility locus associated with the non-coding variant rs12708716. This finding was confirmed immediately for T1D in populations of European descent [20,29]. To date, several SNPs (rs2903692, rs17673553, rs725613, rs12708716, rs12921922, rs12931878) within the CLEC16A gene have been reported to be associated with T1D in several populations: Sardinian [40], Spanish [54], south-east USA [44], Chinese [41,55], and Japanese [56]. Recently CLEC16A was also associated with adult-onset of autoimmune diabetes [57].

Several GWAS in different autoimmune diseases such as multiple sclerosis [40,58,59], primary adrenal insufficiency [60], systemic lupus erythematosus [61,62], Celiac disease [63], Crohn’s disease [64], selective immunoglobulin A deficiency [65], alopecia areata [65], juvenile idiopathic arthritis [66], rheumatoid arthritis [54,66], and primary biliary cirrhosis [67,68] also demonstrated association of the 16p13 loci with disease risk, implying that the 16p13 region contains a key regulator of the self-reactive immune response.

Recently, Davison *et al.* reported intron 19 of the CLEC16A gene behaves as a regulatory sequence, which affects the expression of a neighboring gene dexamethasone-induced (DEXI) [69]. While it is clear that intron 19 of CLEC16A is highly enriched for transcription-factor-binding events, more functional studies are needed to advance from GWAS to candidate causal genes and their biological functions.

Little is yet proven about CLEC16A functions. Kim *et al.*, 2010 characterized an endosomal membrane protein “ema” to be required for endosomal trafficking and promotes endosomal maturation in fruit flies [70]. Expression of human orthologue of ema “CLEC16A” rescued the Drosophila mutant demonstrating conserved function of the protein. A more recent study by the same group also reported its requirement for the growth of autophagosomes and proposed that the Golgi is a membrane source for autophagosomal growth, and that ema facilitates this process [71]. Expression of CLEC16A rescued the autophagosome size defect in the ema mutant, suggesting that regulation of autophagosome morphogenesis may be one of the fundamental functions of CLEC16A. Another study elucidated the dynamic expression changes and localization of CLEC16A in lipopolysaccharide (LPS) induced neuroinflammatory processes in adult rats. CLEC16A expression was strongly induced in active astrocytes in inflamed cerebral cortex. *In vitro* studies indicated that the up-regulation of CLEC16A may be involved in astrocyte activation following LPS challenge [72].

CLEC16A is well-established T1D susceptibility gene, which probably contributes to the disease by modulating immunity and thus the encoded protein, is of high interest for further functional studies.

#### 2.5.2. Latest Novel T1D Susceptibility Loci (2011–2013)

In our latest effort to identify additional genetic loci for T1D, we examined associations in the largest meta-analysis to date between T1D and ~2.54 million SNPs in a combined cohort of 9,934 cases and 16,956 controls. Targeted follow-up of 53 SNPs in 1,120 affected trios uncovered three novel loci associated with T1D that reached genome-wide significance [38].

##### 2.5.2.1. Region 13q22

The most significantly associated SNP (rs539514, *p* = 5.66 × 10^−11^) resides in an intronic region of the LMO7 (LIM domain only 7) gene on 13q22 [38]. LMO7 is a multi-domain mammalian protein with a calponin homology (CH) domain, a discs-large homologous regions (DHR) domain, and a LIM domain. Proteins of this family are involved in protein-protein interactions, regulation of cell adhesion and signaling [73,74]. The expression of LMO7 is cell type specific and is essential for the development of muscle and heart tissues [75,76,77]. Mice with homozygous deletions of LMO7 display retinal, muscular, and growth retardation [78]. LMO7 is known to be upregulated in multiple cancers, especially at the metastatic stage [79]. In cultured rat ascites hepatoma cells, the upregulation of LMO7 correlates with the ability of transforming growth factor β (TGFβ) to enhance the invasiveness of these cells [80]. Recent GWAS meta-analysis from our group identified LMO7 association with T1D [38]. Although the function of LMO7 does not clearly relate to the etiology of T1D, LMO7 is expressed in pancreatic islets and thus is a plausible biological candidate at this locus [81].

##### 2.5.2.2. Region 2q23

The second most significantly associated SNP (rs478222, *p* = 3.50 × 10^−9^) resides in an intronic region of the EFR3B (protein EFR3 homolog B) gene on 2p23; however, the region of linkage disequilibrium is approximately 800 kb and harbors additional multiple genes, including NCOA1, C2orf79, CENPO, ADCY3, DNAJC27, POMC, and DNMT3A. Protein EFR3B is an 817 amino acid and exists as three alternatively spliced isoforms. The gene encoding EFR3B maps to human chromosome 2p23.3. A number of genetic diseases have been linked to genes on chromosome 2 including Harlequin icthyosis [82], lipid metabolic disorder sitosterolemia [83], and Alstrom syndrome [84]. Our recent study showed novel association of 2q23 locus with T1D risk [38]. Though the 2q23 region harbors additional multiple genes, including NCOA1, C2orf79, CENPO, ADCY3, DNAJC27, POMC, and DNMT3A, location of SNP rs478222 in the intronic region of EFR3B makes it a good candidate gene.

Nuclear receptor coactivator 1 protein (NCOA1) is a member of the p160/steroid receptor co-activator (SRC) family. The product of this gene binds to a variety of nuclear hormone receptors in a ligand-dependent manner suggesting that NCOA1 may play a role as a bridging molecule between nuclear hormone receptors and general transcription factors [85,86].

C2orf79 is peptidyl-tRNA hydrolase domain containing 1 (PTRHD1) predicted protein with unknown function.

Centromere protein O gene (CENPO) encodes a component of the interphase centromere complex. The protein is localized to the centromere throughout cell division and is required for bipolar spindle assembly, chromosome segregation and checkpoint signaling during mitosis [87].

Adenylate cyclase 3 gene (ADCY3) encodes a membrane-associated enzyme. This protein catalyzes the formation of the secondary messenger cyclic adenosine monophosphate (cAMP) and is highly expressed in human placenta, testis, ovary, and colon [88]. Expression of adenylyl cyclase 2, 3, and 4 has been reported in olfactory cilia; ADCY3 mutants failed olfaction-based behavioral tests indicating that ADCY3 and cAMP signaling are critical for olfactory-dependent behavior [89].

DnaJ/Hsp40 homolog, subfamily C, member 27 gene (DNAJC27) encodes 273 amino acid protein with RAB-like GTPase and DNAJ domains. EST database reports high expression in nervous and reproductive systems [90].

Pro-opiomelanocortin gene (POMC) encodes a polypeptide hormone precursor protein synthesized mainly in corticotroph cells of the anterior pituitary. POMC is essential for normal steroidogenesis and maintenance of adrenal weight. Mutations in this gene have been associated with early onset of obesity, adrenal insufficiency, and red hair pigmentation [91,92]. The recent study in UK population suggested that POMC SNP haplotype GGCGAG may have a protective effect against T1D [93].

DNA (cytosine-5)-methyltransferase 3 alpha gene (DNMT3A) encodes a protein that functions as a *de novo* methyltransferase that can methylate unmethylated and hemimethylated DNA with equal efficiencies [94].

Additional fine gene mapping and functional studies are needed for above-mentioned genes to determine causal variants for 2q23 region and their role in T1D.

##### 2.5.2.3. Intergenic Region 6q27

Intergenic region on 6q27 contained the third most significantly associated SNP (rs924043, *p* = 8.06 × 10^−9^) in our recent study [38]. The region of association is approximately 900 kb and harbors multiple genes including PHF10, TCTE3, DLL1, FAM120B, PSMB1, TBP, and PDCD2. The 6q27 region also includes several genes of unknown function: C6orf208/LINC00574 (long intergenic non-protein coding RNA 574), T-complex-associated-testis-expressed 3 (TCTE3), LOC154449, WD repeat domain 27 (WDR27), and chromosome 6 open reading frame 120 (C6orf120).

Plant Homeo Domain (PHD) finger protein 10 gene (PHF10) encodes a subunit of an ATP-dependent chromatin-remodeling complex that functions in neural precursor cells [95].

Delta-like 1-Drosophila gene (DLL1) is a human homolog of the Notch Delta ligand and a member of the delta/serrate/jagged family. It plays a role in mediating cell fate decisions during hematopoiesis and cell communication [96,97]. The protein is expressed in heart, pancreas and brain. Pancreatic regeneration in chronic pancreatitis requires activation of the notch signaling pathway [98].

The family with sequence similarity 120B gene (FAM120) encodes protein belonging to the constitutive coactivator of peroxisome proliferator-activated receptor gamma (PPARG) family. FAM120B functions in adipogenesis through PPARG activation in a ligand-independent manner [99].

Proteasome (prosome, macropain) subunit, beta type, 1 gene (PSMB1) encodes a member of the proteasome B-type family, also known as the T1B family, that is a 20S core beta subunit [100]. This gene encodes TBP, the TATA-binding protein, transcription factor that functions at the core of the DNA-binding multiprotein transcription factor IID (TFIID). Binding of TFIID to TBP is the initial transcriptional step of the pre-initiation complex (PIC) and plays a role in the activation of eukaryotic genes transcribed by RNA polymerase II [101].

Programmed cell death 2 gene (PDCD2) encodes a nuclear protein highly expressed in placenta, heart, pancreas, lung, and liver, and lowly expressed in spleen, lymph nodes, and thymus. Expression of this gene is shown to be repressed by B-cell CLL/lymphoma 6 (BCL6), a transcriptional repressor [102].

In addition, despite not reaching the genome wide significance, our study observed evidence for association at three additional loci containing the candidate genes LOC100128081, TNFRSF11B, and FOSL2 [38]. Of these, it is notable that the tumor necrosis factor receptor superfamily, member 11B (TNFRSF11B) is a strongly associated locus with bone mineral density, also discovered in GWAS, and the locus harboring LOC100128081 has also been reported in the context of a GWAS of SLE. FOS-like antigen 2 (FOSL2) gene encodes a leucine zipper protein that dimerizes with the JUN family proteins and forms the transcription factor complex activator protein 1 (AP-1). The FOS proteins have been implicated as regulators of cell proliferation, differentiation, and transformation [103].

##### 2.5.2.4. Region 12q24

CUX2 (12q24): Huang *et al.*, 2012 re-analyzed the original 2007 WTCCC study by using the 1,000 Genomes imputation and reported refined variant rs1265564 in Cut-like homeobox 2 (CUX2) region for association with T1D [46]. CUX2 is expressed exclusively in neural tissues. The protein belongs to the CUT homeobox family and contains three CUT domains and a homeodomain, both domains are DNA-binding motifs [104]. CUX2 gene has been shown to directly regulate the expression of NeuroD [105]. NeuroD/BETA2, a transcription factor of the insulin gene, is reported to be associated with T1D in Asian descent [106,107]. Thus, CUX2 is a plausible candidate for exploration in T1D pathogenesis.

##### 2.5.2.5. Region 5p13-q13

HTR1A (5p13-q13): Asad *et al.* confirmed [45] the previously suggested association between the chromosome 5p13-q13 region and T1D in Scandinavian families [108]. None of the previous GWAS have reported any association of 5p13-q13 with T1D. This recent study identified the 5-hydroxytryptamine receptor 1A (HTR1A), and the ring finger protein 180 (RFN180) genes, to be associated with T1D in multiplex (Swedish and Danish) families. However, the conditional analysis indicated HTR1A has as a primary association with T1D. Both quantitative PCR and immunohistochemical analysis confirmed the presence of the HTR1A in human pancreas [45]. The study suggests that HTR1A may affect T1D susceptibility by modulating the initial autoimmune attack or either islet regeneration, insulin release, or both. The HTR1A gene is known to encode for a G-protein coupled receptor specific for serotonin, which mediates cellular signaling via the amine serotonin [109]. The HTR1A receptor is mainly known to mediate signal transduction in neurons in the central nervous system [110]. However, serotonin is also produced in pancreatic islets of several different species [111]. Studies in rodent islets show inhibition of insulin secretion by serotonin [112]. Sumatriptan (serotonin agonist) has an inhibitory effect on insulin secretion in humans [113]. Previously a decrease in expression of HTR1A with increased insulin release during pancreatic regeneration has been reported [114]. HTR1A also plays a role in the immune system by downregulating adenylate cyclase, which in turn regulates T-cell cytokine production and cytotoxicity [115]. Hence, polymorphisms in the HTR1A gene may affect insulin release and T-cell activity thereby increases the risk of developing T1D.

## 3. Conclusions

This review provides a summary of recent advances in the identification of risk variants associated with T1D. Genome wide association studies have revolutionized the discovery approach to autoimmune mediated disorders. In T1D only six genetic factors were well known before GWAS. GWAS has contributed greatly by expanding the number of established genetic variants to 59 loci. Most of these genes are novel and were not in any investigator’s favorite list. For the first time there is real consensus on the role of specific genetic factors underpinning T1D pathogenesis.

The discoveries of genetic factors involved in T1D through GWAS present the first step in a long process leading to cure. Genes uncovered using this approach are indeed fundamental to disease biology and will define the key molecular pathways leading to cure of T1D. However, such genome wide scans can lack coverage in certain regions where it is difficult to genotype, thus, it is possible that other loci with reasonable effect sizes remain to be uncovered through whole genome sequencing approaches.

To date most of T1D associated variants have been discovered utilizing cohorts of European ancestry because the SNP arrays were designed to optimally capture the haplotype diversity in this ethnicity. Novel SNP arrays are needed with the same degree of capture in diverse populations to elucidate the full role of each locus in a worldwide context.

In addition to identifying genes influencing disease susceptibility GWAS can be utilized to facilitate implementation of personalized medicine based on genetic make-up of the individuals. Our pilot study showed a proof-of-principle that use of whole-genome data, rather than a few ‘‘validated’’ susceptibility loci, could improve predictive accuracy [116]. This approach will have a greater impact on health care in the future; for example, by applying personalized intervention strategies on newborns who are at risk of developing T1D, we may reduce their risk of developing the disease or be better prepared to treat the disease.

The next challenge is to resolve the specific causal variants and determine how they affect the expression and function of these gene products. The Next-Generation Sequencing (NGS) technology has opened new avenues to elucidate the role of coding and noncoding RNAs in health and disease and is speeding up the identification of causative gene variants in T1D. 

No doubt, the *in vitro* and *in vivo* biology of these genes will be fascinating areas of exploration for many scientists. Only after scientists have fully uncovered the functional context of T1D associated genes, is the promise of new therapies and preventive strategies likely to materialize.
